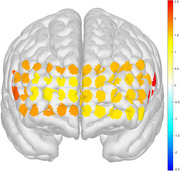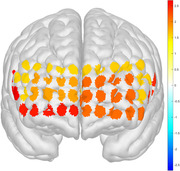# Prefrontal Hemodynamic Activity during Working Memory Challenges: Investigating Stimulus Influence

**DOI:** 10.1002/alz.086007

**Published:** 2025-01-03

**Authors:** Seungju Lim, Ji‐Hyuk Park

**Affiliations:** ^1^ Yonsei University, Wonju, Gangwon‐do Korea, Republic of (South)

## Abstract

**Background:**

Research on hemodynamic responses in the prefrontal cortex during working memory tasks has mainly been performed in cognitive neuroscience research. However, the specific stimuli contributing to working memory deficits in major depressive disorder (MDD) are currently lacking. This study aims to compare working memory performance and prefrontal cortex activation in individuals with MDD and healthy controls during working memory tasks involving textual, visual, and emotional stimuli.

**Method:**

Functional near‐infrared spectroscopy was utilized to record hemodynamic responses from 71 adults (30 with MDD and 41 controls) during the 2‐back tasks with four stimulus types: numbers, letters, polygons, and facial expressions. Working memory performance was measured by accuracy and reaction time during the tasks, and hemodynamic responses were collected by oxyhemoglobin (HbO) activation of the prefrontal cortex during the tasks.

**Result:**

MDD demonstrated lower working memory performance than controls during polygon and facial expression‐based 2‐back tasks, while there was no significant difference under number and letter conditions. MDD under the polygon stimulus exhibited abnormal HbO activation in the bilateral ventrolateral prefrontal cortex compared to controls, while under the facial expression stimulus, it showed exclusive abnormal HbO activation in the left medial prefrontal cortex, bilateral orbito prefrontal cortex, and bilateral ventrolateral prefrontal cortex.

**Conclusion:**

These findings indicate the importance of stimuli that require cognitive processing not only for logical aspects, such as polygons, but also for emotional aspects, like facial expressions, in evaluating hemodynamic responses of prefrontal cortex and working memory abilities in individuals with MDD.